# UPLC-MS/MS Method for the Simultaneous Quantification of Eight Compounds in Rat Plasma and Its Application to a Pharmacokinetic Study after Oral Administration of Veratrum (*Veratrum nigrum* L.) Extract

**DOI:** 10.1155/2020/8883277

**Published:** 2020-10-19

**Authors:** Yuqi Fan, Lulu Zhao, Xuhua Huang, Jiayuan Shen, Wei Wang, Xiaohua Jia, Mengyuan Gao, Huizi Ouyang, Yanxu Chang, Jun He

**Affiliations:** ^1^First Teaching Hospital of Tianjin University of Traditional Chinese Medicine, Tianjin 301617, China; ^2^Tianjin State Key Laboratory of Modern Chinese Medicine, Tianjin University of Traditional Chinese Medicine, Tianjin 301617, China

## Abstract

*Veratrum nigrum* L. is a well-known traditional Chinese medicine with a lot of pharmacological activities including antihypertensive, anticancer, and antifungal effects. In the current experiment, a rapid and sensitive UPLC-MS/MS method that takes only 7 min run time has been established and validated for simultaneous determination of eight bioactive compounds including cyclopamine, jervine, veratramine, polydatin, quercetin, apigenin, resveratrol, and veratrosine in rat plasma. The chromatographic separation of analytes and internal standard was performed on a Phenyl-Hexyl column (2.1 × 100 mm, 1.7 *μ*m) with the mobile phase consisting of water (0.1% formic acid) and acetonitrile at a flow rate of 0.3 mL/min. An electrospray ionization (ESI) source was used to detect the samples in both positive and negative ion modes. The intra- and interday precisions of the compounds were less than 9.5% and the accuracy ranged from -10.8% to 10.4%. The extraction recoveries of the compounds were in the range of 85.1 ± 1.5% to 102.6 ± 8.0%, and the matrix effect ranged from 91.2 ± 4.5% to 113.8 ± 1.5%. According to the results of the stability test, the eight compounds have good stability under various conditions and the relative standard deviation (RSD) less than 13.2%. The pharmacokinetic parameters of the eight compounds in rat plasma after oral administration of *Veratrum nigrum* L. extract were successfully determined by the established UPLC-MS/MS method.

## 1. Introduction

*Veratrum nigrum* L., known as “LiLu”, is a famous traditional Chinese medicine that consists of the dried rhizomes and roots of *Veratrum nigrum* L. [1]. Despite its well-known toxic properties, it has thousands of years of medicinal history in China, and during the Middle Ages, it has been used for medical purposes in Europe [2, 3]. It has been used for treatment of epilepsy, excessive phlegm, blood-stroke, and hypertension [4], while at the same time, is known to have mutagenic potential, hepatotoxicity, skeletal and neurotoxicity, and muscle toxicity [5–7]. The main components of Veratrum are alkaloids and stilbene [8, 9]. Pharmacological and chemical studies have been revealed that the major toxic and bioactive compounds of this traditional Chinese medicine are steroidal alkaloids [10]. More and more pharmacological studies proved that various ingredients in *Veratrum nigrum* L. have a lot of pharmacological activities such as antithrombotic activity, anticancer, *β*2-adrenoceptor agonist, antihypertensive, and antifungal effects. It also prevents obesity and alleviates allergy and contact dermatitis etc. [11–18].

Since traditional Chinese medicine has become more and more popular throughout the world in view of its curative effects and efficacy, it is indispensable to figure out the pharmacokinetic changes of its active ingredients in the body. As we all know, the efficacy of traditional Chinese medicine is generally based on the synergy of various ingredients. Therefore, it is essential to simultaneously determine the main bioactive components in biological samples in regard to multicomponent pharmacokinetic studies. Up to now, the reported pharmacokinetic research of the components in *Veratrum nigrum* L. is mainly focused on the components of higher content such as jervine, resveratrol, and veratramine [19–24]. Therefore, it is necessary to further study the pharmacokinetics of various ingredients in rat plasma after oral administration of *Veratrum nigrum* L. extract.

UPLC-MS/MS has the characteristics of high sensitivity and accuracy, and it is well known that UPLC-MS/MS plays a significant role in the quantification of the main components of medicinal products for pharmacokinetic studies [25–27]. In this study, a rapid and sensitive UPLC-MS/MS method was firstly established and applied to simultaneously quantify eight bioactive compounds (cyclopamine, jervine, veratramine, polydatin, quercetin, apigenin, resveratrol, and veratrosine) in rat plasma after oral administration of *Veratrum nigrum* L. extract. This study would provide some references for further pharmacology and pharmacodynamics studies of *Veratrum nigrum* L. extract.

## 2. Experimental

### 2.1. Reagents and Chemicals

**Figure 1:**
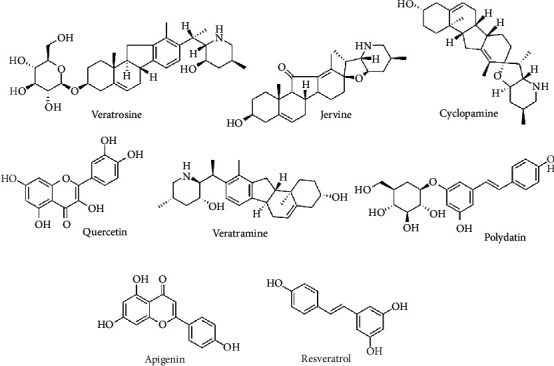
The chemical structures of the eight compounds.

Methanol and acetonitrile (chromatographic purity) were purchased from Merck KGaA (Darmstadt, Germany). Formic acid (chromatographic purity) was obtained from ROE (St. Louis, MO, USA). A Milli-Q water purification system (Millipore, Milford, MA, USA) was used for preparing the ultrapure water. Cyclopamine, jervine, veratramine, polydatin, quercetin, apigenin, resveratrol, and hupehenine (internal standard, IS) were purchased from Chengdu Must Bio-Technology Co., Ltd (Chengdu, China). Veratrosine was purchased from Chengdu Chroma-Biotechnology Co., Ltd. (Chengdu, China). The chemical structures of the analytes are displayed in Figure 1.

### 2.2. Instruments and Conditions

An Agilent 1290 ultraperformance liquid chromatography coupled to an Agilent 6470 series triple quadrupole mass spectrometer with an Agilent Jet Stream (AJS) ESI source was used for analyzing all samples of this study. 0.1% formic acid in water (A) and acetonitrile (B) were used as mobile phases with the following gradient elution method: 0-2 min, 15%-25% B; 2-4 min, 25%-50% B; 4-6 min, 50%-90% B; and 6-7 min, 90%-95% B. The separation was performed on a Waters ACQUITY UPLC^®^ CSHTM Phenyl-Hexyl (2.1 × 100 mm, 1.7 *μ*m) column, and the temperature was maintained at 20°C. The flow rate and injection volume were 0.3 mL/min and 5 *μ*L, respectively. Quantitative parameters are recorded in Table 1.

### 2.3. *Veratrum nigrum* L. Extract Preparation

To prepare the *Veratrum nigrum* L. extract, 200 g dried rhizome of *Veratrum nigrum* L. plant was accurately weighed and extracted by refluxing with 70% (*v*/*v*) ethanol (ratio of 1 g: 15 mL); repeat three times for two hours each time. After that, the obtained extracts were filtered and mixed, and then the solvent was evaporated under reduced pressure (50°C, 100 hPa). Finally, the dried *Veratrum nigrum* L. extracts were pulverized into fine powders and stored in a desiccator until analysis. The contents of veratrosine, jervine, cyclopamine, veratramine, polydatin, quercetin, apigenin, and resveratrol in *Veratrum nigrum* L. extract powder were 9.4, 7.8, 5.4, 89.7, 74.3, 0.5, 0.6, and 2.7 *μ*g/g, respectively.

### 2.4. Preparation of Standard Solutions, Calibration Standards, and Quality Control Samples

Cyclopamine, jervine, veratramine, polydatin, quercetin, apigenin, resveratrol, veratrosine, and hupehenine (internal standard, IS) were separately weighed and dissolved with methanol as standard stock solutions (1 mg/mL). The calibration solutions were prepared by adding appropriate volumes of mixture working solution and 20 *μ*L of IS into 100 *μ*L blank rat plasma, resulting in concentrations: 2.4, 4.8, 12, 24, 60, 120, 300, and 600 ng/mL for veratrosine; 4, 8, 20, 40, 100, 200, 500, and 1000 ng/mL for jervine; 0.4, 0.8, 2, 4, 10, 20, 50, and 100 ng/mL for cyclopamine, polydatin, quercetin, and apigenin; 1.6, 3.2, 8, 16, 40, 80, 200, and 400 ng/mL for veratramine; and 0.8, 1.6, 4, 8, 20, 40, 100, and 200 ng/mL for resveratrol. Quality control (QC) samples at three levels (low, medium, and high concentrations) were prepared in the same manner. All the samples were kept at 4°C until analysis.

### 2.5. Preparation of Plasma Sample

100 *μ*L plasma sample was incorporated with 20 *μ*L methanol and 20 *μ*L of IS (20 ng/mL), and vortexed for 30 seconds. 1 mL ethyl acetate was then added to the sample and vortex-mixed for 3 min. The samples were centrifuged (12000 × *g*, 10 min, 4°C), and the supernatant was carefully collected to a clean tube and evaporated to dryness under a nitrogen stream. The obtained residue was reconstituted in 100 *μ*L methanol and centrifugation at 12000 × *g* for 10 min. 5 *μ*L of the supernatant was injected for UPLC-MS/MS measurement.

### 2.6. Method Validation

#### 2.6.1. Specificity

The specificity was carried out by comparing chromatograms of blank rat plasma samples, with blank plasma spiked with analytes and postdosing plasma samples gained from the rats given oral administration of *Veratrum nigrum* L. extract to evaluate any potentially endogenous plasma matrix components that could result in chromatographic interference.

#### 2.6.2. Linearity and Sensitivity

We constructed a calibration curve by analyzing the mixed standard solution and IS calibration samples to assess the linearity. The calibration curve equation was calculated using the peak area ratios of analytes against the internal standard as the standard concentration. A linear regression equation with weight coefficient 1/*x*^2^ was used to describe the regression relationship. The limit of quantification (LOQ) was used to assess sensitivity of the method.

#### 2.6.3. Precision and Accuracy

Intraday and interday accuracy and precision were assessed by preparing and analyzing six duplicate quality control (QC) samples with three certain concentration levels (4.8, 60, and 600 ng/mL for veratrosine; 1.6, 20, and 200 ng/mL for resveratrol; 8, 100, and 1000 ng/mL for jervine; 3.2, 40, and 400 ng/mL for veratramine; and 0.8, 10, and 100 for cyclopamine, polydatin, quercetin, and apigenin) within 1 day or on 3 consecutive days. Accuracy was determined by the relative error (RE) value, while the intra- and interday precision was defined as the relative standard deviation (RSD).

#### 2.6.4. Extraction Recovery and Matrix Effect

The matrix effect and extraction recovery were determined with six replicates of QC samples. The extraction recovery was investigated by comparing the peak areas of the eight compounds using QC samples of three concentrations and the peak areas of the postextraction spiked samples. Matrix effect was investigated by comparing the peak areas of postextraction spiked samples with the peak areas of the corresponding working solutions on three QC levels to assess the matrix effect of the samples.

#### 2.6.5. Stability

The QC samples of three concentration levels were placed under various conditions including being stored at autosampler for 12 h, under three freeze-thaw cycles, at room temperature for 6 h and stored at -80°C for 7 days to study the stability of the samples.

### 2.7. Pharmacokinetic Studies

Six male Sprague-Dawley rats (body weight, 220 ± 10 g) were used for this experiment. The rats had free access to water and fasted for 12 h prior to the study. 0.5% CMC-Na aqueous solution was used to dissolve the *Veratrum nigrum* L. extract to a concentration of 0.2 g/mL suspension. The rats were orally administered at a dose of 2 g/kg with the suspension, and approximately 220 *μ*L blood samples was obtained from the ophthalmic venous plexus into centrifuge tubes at predose, and 0.03, 0.08, 0.17, 0.25, 0.5, 1, 2, 4, 6, 8, 10, 12, 24, 36, 48, 60, 72, 84, 96, 108, and 120 h postadministration in rats. After centrifugation at 4000 × *g* for 10 min at 4°C, the collected plasma was transferred into clean tubes and frozen at -80°C until next analysis. Pharmacokinetic parameters were calculated using “Drug and Statistics 3.0” (DAS 3.0) (Medical College of Wannan, China). The animal protocol was approved by the Animal Ethics Committee of Tianjin University of Traditional Chinese Medicine (TCM-LAEC20190060).

## 3. Result and Discussion

### 3.1. Optimization of UPLC-MS/MS Conditions

To achieve better separation of the eight compounds well, UPLC conditions consisting of the column type as well as the proportion and composition of the mobile phase were optimized since they make a vast difference in achieving the goals. This experiment investigated the influence of chromatographic columns CORTECS^®^ UPLC^®^ C_18_ column (2.1 mm × 100 mm, 1.6 *μ*m) and ACQUITY UPLC^®^ CSHTM Phenyl-Hexyl column (2.1 × 100 mm, 1.7 *μ*m) on chromatographic peaks and elution time. The results showed that the ACQUITY UPLC^®^ CSHTM Phenyl-Hexyl column (2.1 × 100 mm, 1.7 *μ*m) had better separation and sensitivity for the eight compounds, and the analysis time was shorter. Meanwhile, the matrix effect and extraction recovery results were more in line with the experimental requirements. The CORTECS^®^ UPLC^®^ C_18_ column (2.1 mm × 100 mm, 1.6 *μ*m) is prone to compound residues during the analysis, which could affect the accuracy of the analysis. Therefore, the former was chosen as the analytical column in the study. The results showed that 0.1% formic acid/water-acetonitrile was the best choice of mobile phase for this study, when compared to methanol-0.1% formic acid/water or methanol-water as it resulted in satisfactory peak shapes of all eight compounds.

The selected internal standard hupehenine is a form of steroidal alkaloids, which is the same type of compound as the main compounds of *Veratrum nigrum* L. extract. It has a good response in positive ion mode and is a nonendogenous substance. The extraction recovery of hupehenine is stable and the peak time is suitable. Mass spectrometry parameters are optimized in the mass spectrometer to enable the compounds to have a better response. The optimized MS parameters were set according to the following: drying gas flow rate at 11 L/min, capillary voltage at 4000 V, temperature at 320°C, and nebulizer at 15 psi.

### 3.2. Optimization of Pretreatment Procedure

Preparation of sample with an appropriate method is a critical step in pharmacokinetic analysis. We tested liquid-liquid extraction (LLE) and protein precipitation (PPT) methods and compared the effects of these two methods on sample pretreatment. The results demonstrated that of the two methods, LLE with ethyl acetate has the characteristics of better recovery and efficiency of the eight compounds compared with PPT extraction with acetonitrile and methanol. Considering these factors, ethyl acetate extraction method was selected to be the sample pretreatment method of this study.

### 3.3. Method Validation

#### 3.3.1. Specificity

**Figure 2:**
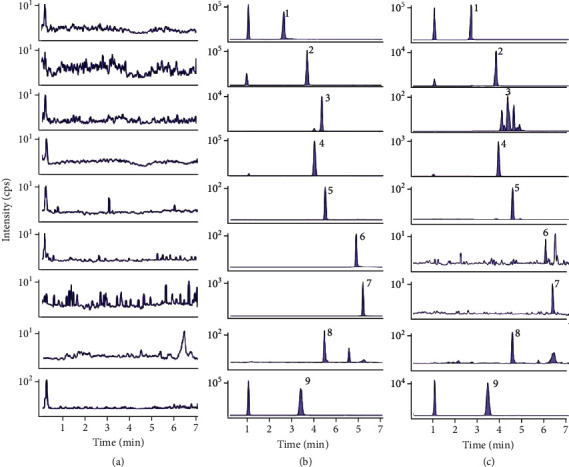
MRM chromatograms of veratrosine (1), jervine (2), cyclopamine (3), veratramine (4), polydatin (5), quercetin (6), apigenin (7), resveratrol (8), and hupehenine (9). The chromatograms of blank plasma (a), blank plasma samples spiked with compounds and IS (b), and plasma sample after oral administration of *Veratrum nigrum* L. extract (c).

Typical chromatograms of blank plasma (a), blank plasma incorporated with eight analytes and IS (b), and plasma samples after dosing (c) are illustrated in Figure 2. No endogenous interference was found in the samples.

#### 3.3.2. Linearity and Sensitivity

Table 2 summarizes the LOQs and calibration curves in rat plasma. The plasma calibration curves exhibited a good linear relationship within the range of 4-1000 ng/mL for jervine; 0.4-100 ng/mL for cyclopamine, polydatin, quercetin, and apigenin; 1.6-400 ng/mL for veratramine; 0.8-200 ng/mL for resveratrol; and 2.4-600 ng/mL for veratrosine. Correlation coefficients of the eight compounds were higher than 0.9902. And the LOQ of jervine was a little higher than the others. It may be caused by the correspondingly higher interference in the blood matrix, making the background noise increase. The LOQs of the eight compounds were less than 4 ng/mL, indicating that the sensitivity was sufficient for pharmacokinetic analysis.

#### 3.3.3. Precision and Accuracy

The accuracy and intraday and interday precision were assessed according to the RE and RSD values.  Table 3 sums up the data of the accuracy and intra- and interday precision. As shown in Table 3, RE values of average accuracy ranged from −10.8% to 10.4%, and the RSD values were less than 9.5% for intra- and interday precision.

#### 3.3.4. Matrix Effect and Extraction Recovery

The mean recovery rates of the eight compounds were ranged from 85.1 ± 1.5% to 102.6 ± 8.0%, and the mean matrix effect ratio for all analytes was ranged from 91.2 ± 4.5% to 113.8 ± 1.5% (Table 4). The results indicated that the influence from plasma matrix was not worth mentioning.

#### 3.3.5. Stability

This experiment investigated different stabilities of the QC samples at three concentration levels through the following four conditions: stored at autosampler for 12 h after preparation (postpreparation stability), at room temperature for 6 h (short-term stability), at three freeze-thaw cycles (free-thaw stability), and at -80°C for 7 days (long-term stability). The RSD value of the replicate QC samples was less than 13.2% as listed in Table 5. The data suggest that the analytes have good stability in the above conditions.

### 3.4. Pharmacokinetic Studies

**Figure 3:**
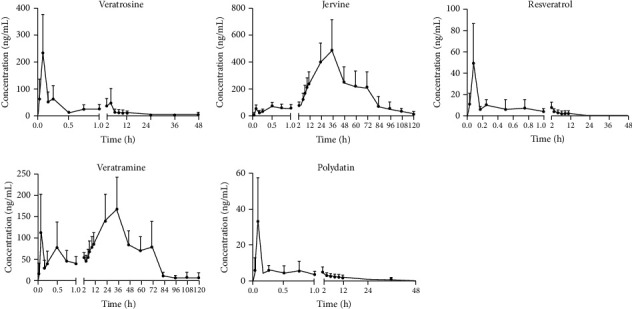
Mean plasma concentration-time curves of veratrosine, jervine, veratramine, polydatin, and resveratrol after oral administration of *Veratrum nigrum* L. extract (mean ± SD, *n* = 6).

The validated analytical method was applied to study the plasma samples obtained from the rats which were intragastric administrations of *Veratrum nigrum* L. extract. The calculated pharmacokinetic parameters are listed in Table 6, and Figure 3 shows the mean plasma concentration-time profile of the bioactive ingredients.

Some important pharmacokinetic parameters were calculated in this study including *C*_max_, the maximum plasma concentration and time to reach *C*_max_ of each compound (*T*_max_). The *T*_1/2_ was the elimination half-life and it reflects the elimination rate of the drug in the body. The area under the plasma concentration-time curve AUC_(0 − *t*)_ was calculated using the trapezoidal rule and extrapolated to infinity for AUC_(0 − ∞)_. The mean residence time (MRT) of these ingredients was also investigated.

In the study, after oral administration of *Veratrum nigrum* L. extract, cyclopamine, quercetin, and apigenin in rat plasma were only detected at the first few plasma sampling points, which made it difficult to form a complete pharmacokinetic curve; hence, we excluded the three analytes in the following results.

As shown in Table 6, the *C*_max_ of jervine at 515.0 ± 242.4 ng/mL showed that the blood concentration of jervine was higher than the other compounds and its absorption was more complete. The *T*_max_ of veratrosine, polydatin, and resveratrol were 0.08 ± 0.00 h, 0.26 ± 0.29 h, and 0.18 ± 0.17 h, respectively, showing that the absorption of these three compounds was rapid. The *T*_max_ of jervine and veratramine were both longer than 32 h, demonstrating that these two components were slowly absorbed in the rats, which is consistent with the chronic toxicity of *Veratrum nigrum* L. reported in literatures and is also consistent with the death time of rats in the preliminary experiments [4, 28].

The AUC_(0 − *t*)_ and the AUC_(0 − ∞)_ of all five compounds were close by indicating that the monitoring time of this study was appropriate. The AUC_(0 − *t*)_ of jervine was larger than other analytes, showing an abundant of plasma exposure higher than other compounds. The *T*_1/2_ of resveratrol was 2.24 ± 1.11 h, which was faster when compared to other compounds. Jervine and veratramine were components that caused the toxicity of *Veratrum nigrum* L. [7]. The long half-life of the terminal phase and the long mean residence time of jervine and veratramine may explain the phenomenon and causes of chronic toxicity of *Veratrum nigrum* L., which may provide a theoretical basis for further studies.

To sum up the all pharmacokinetic results, we can find that the veratrosine, polydatin, and resveratrol can be absorbed into the blood faster while jervine and veratramine were slowly absorbed. Compared with other ingredients, jervine is more completely absorbed and widely distributed and has a longer residence time in the body. And resveratrol has the fastest metabolizing speed among the five ingredients.

## 4. Conclusion

A rapid and sensitive UPLC-MS/MS method was developed to determine the eight compounds (cyclopamine, jervine, veratramine, polydatin, quercetin, apigenin, resveratrol, and veratrosine) after oral administration of *Veratrum nigrum* L. extract in rats' plasma in the experiment. This method is specific, stable, and reliable and has a short analysis time. The results indicated that the blood concentration and plasma exposure of jervine were higher than other compounds. In addition, the absorption of veratrosine, polydatin, and resveratrol was quick. Jervine and veratramine have longer terminal phase half-life, and the longer mean residence time compared to other compounds might be the reason for *Veratrum nigrum* L.'s chronic toxicity. The pharmacokinetic parameters provided valuable information for the further development and clinical application of *Veratrum nigrum* L.

## Figures and Tables

**Table 1:** Mass spectra properties of eight analytes and IS.

Compounds	Precursor ion (m/z)	Product ion (m/z)	Frag. (V)	C.E. (V)	Ion mode
Veratrosine	572.4	457.2	242	40	Positive
Jervine	426.3	114.0	214	36	Positive
Cyclopamine	412.3	114.0	80	36	Positive
Veratramine	410.3	295.1	189	32	Positive
Polydatin	389.1	227.0	156	28	Negative
Quercetin	301.0	151.0	128	24	Negative
Apigenin	269.0	117.0	141	40	Negative
Resveratrol	229.1	107.0	93	24	Positive
Hupehenine	416.4	98.1	272	60	Positive

**Table 2:** Calibration curves, correlation coefficients, linear ranges, and LOQ of the 8 analytes.

Compounds	Calibration curves	Correlation coefficients (*r*^2^)	Linear range (ng/mL)	LOQ (ng/mL)
Veratrosine	*Y* = 0.3932*X* + 0.0043	0.9924	2.4-600	2.4
Jervine	*Y* = 0.3625*X* + 0.0021	0.9971	4.0-1000	4.0
Cyclopamine	*Y* = 0.4426*X* + 2.3890*E*^−004^	0.9908	0.4-100	0.4
Veratramine	*Y* = 4.0524*X* + 0.0365	0.9909	1.6-400	1.6
Polydatin	*Y* = 0.0083*X* + 7.1378*E*^−006^	0.9902	0.4-100	0.4
Quercetin	*Y* = 0.0071*X* + 1.096*E*^−006^	0.9902	0.4-100	0.4
Apigenin	*Y* = 0.0203*X* + 3.2596*E*^−005^	0.9978	0.4-100	0.4
Resveratrol	*Y* = 0.0031*X* + 3.9723*E*^−007^	0.9913	0.8-200	0.8

**Table 3:** Precision and accuracy of 8 analytes in rat plasma (*n* = 6).

Compounds	Spiked concentration (ng/mL)	Intraday			Interday		
		Measured concentration (ng/mL)	Accuracy (RE, %)	Precision (RSD, %)	Measured concentration (ng/mL)	Accuracy (RE, %)	Precision (RSD, %)
Veratrosine	4.8	5.3 ± 0.2	10.4	2.8	5.1 ± 0.1	6.2	1.8
	60	54.4 ± 1.1	-9.3	2.1	56.9 ± 1.4	-5.1	2.4
	600	566.2 ± 20.6	-5.6	3.6	589.8 ± 6.7	-1.7	1.1
Jervine	8	8.6 ± 0.3	7.9	3.1	8.6 ± 0.3	7.5	3.0
	100	103.6 ± 1.1	3.6	1.1	100.8 ± 1.2	0.8	1.2
	1000	930.6 ± 32.0	-6.9	3.4	929.1 ± 34.9	-7.1	3.8
Cyclopamine	0.8	0.9 ± 0.0	7.8	2.6	0.8 ± 0.0	5.0	2.4
	10	10.8 ± 0.3	7.5	2.3	10.3 ± 0.3	3.3	2.9
	100	102.6 ± 3.5	2.6	3.4	100.0 ± 2.3	0.0	2.4
Veratramine	3.2	3.3 ± 0.1	4.4	4.2	3.3 ± 0.1	1.6	1.5
	40	40.6 ± 0.5	1.6	1.3	40.0 ± 0.5	0.1	1.2
	400	380.0 ± 14.8	-5.0	3.9	383.5 ± 11.0	-4.1	2.9
Polydatin	0.8	0.8 ± 0.1	0.0	6.3	0.8 ± 0.1	3.8	6.0
	10	9.9 ± 0.4	-1.5	3.9	10.2 ± 0.5	2.0	4.9
	100	99.2 ± 5.2	-0.8	5.3	99.1 ± 3.2	-0.9	3.2
Quercetin	0.8	0.8 ± 0.1	-5.0	7.9	0.8 ± 0.1	2.5	8.5
	10	10.4 ± 0.2	3.8	2.3	10.1 ± 1.0	0.7	9.5
	100	107.7 ± 3.9	7.7	3.6	101.9 ± 4.6	1.9	4.5
Apigenin	0.8	0.7 ± 0.0	-8.8	2.7	0.8 ± 0.1	-3.8	6.5
	10	9.1 ± 0.3	-9.4	2.9	9.4 ± 0.5	-6.4	5.7
	100	89.2 ± 2.3	-10.8	2.6	91.4 ± 1.4	-8.7	1.5
Resveratrol	1.6	1.6 ± 0.1	-0.6	6.3	1.6 ± 0.0	2.5	1.2
	20	19.0 ± 0.6	-4.9	3.0	19.9 ± 0.8	-0.7	4.0
	200	199.6 ± 4.8	-0.2	2.4	202.9 ± 4.5	1.5	2.2

**Table 4:** Matrix effect and extraction recovery of 8 analytes (*n* = 6).

Compounds	Spiked concentration (ng/mL)	Extraction recovery (%)	RSD (%)	Matrix effect (%)	RSD (%)
Veratrosine	4.8	94.9 ± 6.1	6.4	108.9 ± 4.4	4.0
	60	85.3 ± 3.1	3.7	94.4 ± 3.3	3.5
	600	87.3 ± 0.7	0.8	100.8 ± 1.9	1.9
Jervine	8	94.2 ± 2.7	2.9	108.9 ± 4.3	3.9
	100	87.1 ± 2.4	2.8	101.8 ± 4.0	3.9
	1000	87.1 ± 1.6	1.8	107.0 ± 3.1	2.8
Cyclopamine	0.8	95.9 ± 6.0	6.2	108.0 ± 6.5	6.0
	10	86.9 ± 1.4	1.6	95.1 ± 2.3	2.5
	100	85.1 ± 1.5	1.7	104.4 ± 1.4	1.4
Veratramine	3.2	95.9 ± 8.3	8.6	113.8 ± 1.5	1.3
	40	89.4 ± 6.1	6.8	100.9 ± 2.8	2.8
	400	86.6 ± 1.8	2.1	105.2 ± 3.7	3.5
Polydatin	0.8	92.5 ± 2.3	2.5	108.3 ± 6.1	5.6
	10	90.4 ± 1.1	1.2	96.9 ± 2.5	2.6
	100	86.6 ± 1.1	1.3	107.9 ± 1.1	1.0
Quercetin	0.8	102.6 ± 8.0	7.8	106.6 ± 2.6	2.4
	10	89.3 ± 2.4	2.7	108.1 ± 10.7	9.9
	100	94.0 ± 7.0	7.5	108.9 ± 5.8	5.3
Apigenin	0.8	102.1 ± 5.6	5.5	97.5 ± 2.8	2.9
	10	89.9 ± 7.6	8.4	92.6 ± 4.6	5.0
	100	89.3 ± 3.6	4.1	105.7 ± 4.7	4.5
Resveratrol	1.6	101.5 ± 3.1	3.0	103.4 ± 5.4	5.2
	20	85.3 ± 1.4	1.6	91.2 ± 4.5	5.0
	200	87.4 ± 1.9	2.2	106.9 ± 2.3	2.2

**Table 5:** Stability of 8 analytes in rat plasma (*n* = 6).

Compounds	Spiked concentration (ng/mL)	Room temperature for 6 h		Three freeze-thaw cycles		Autosampler for 12 h		-80°C for 7 days	
		Measured concentration (ng mL^−1^)	RSD (%)	Measured concentration (ng mL^−1^)	RSD (%)	Measured concentration (ng mL^−1^)	RSD (%)	Measured concentration (ng mL^−1^)	RSD (%)
Veratrosine	4.8	5.2 ± 0.2	3.5	5.1 ± 0.1	1.4	4.9 ± 0.2	4.1	5.3 ± 0.1	2.7
	60	56.2 ± 1.4	2.5	59.5 ± 1.9	3.2	57.9 ± 0.1	0.1	67.6 ± 0.9	1.3
	600	523.6 ± 3.9	0.8	540.5 ± 16.6	3.1	562.3 ± 2.9	0.5	604.0 ± 14.5	2.4
Jervine	8	8.7 ± 0.0	0.4	8.9 ± 0.2	1.8	8.8 ± 0.2	2.2	8.5 ± 0.6	7.4
	100	100.5 ± 0.2	0.2	99.3 ± 0.9	0.9	104.8 ± 0.2	0.2	98.0 ± 2.1	2.1
	1000	868.7 ± 3.2	0.4	932.1 ± 3.2	0.4	870.6 ± 13.7	1.6	870.3 ± 13.1	1.5
Cyclopamine	0.8	0.9 ± 0.0	3.4	0.9 ± 0.0	2.3	0.9 ± 0.0	2.3	0.9 ± 0.1	9.2
	10	10.2 ± 0.1	1.4	10.2 ± 0.2	1.6	10.4 ± 0.2	1.5	9.6 ± 0.3	3.4
	100	99.2 ± 1.1	1.1	98.0 ± 2.6	2.7	96.2 ± 1.0	1.0	93.7 ± 2.2	2.4
Veratramine	3.2	3.3 ± 0.1	3.3	3.3 ± 0.1	3.7	3.3 ± 0.1	2.2	3.1 ± 0.1	2.9
	40	38.24 ± 0.89	2.3	40.0 ± 0.6	1.5	40.7 ± 0.5	1.1	45.1 ± 0.3	0.7
	400	356.9 ± 7.2	2.0	366.5 ± 9.0	2.5	361.7 ± 6.0	1.7	351.3 ± 3.3	0.9
Polydatin	0.8	0.8 ± 0.1	8.6	0.8 ± 0.1	10.1	0.9 ± 0.0	3.5	0.9 ± 0.1	7.1
	10	9.6 ± 0.1	0.9	10.1 ± 0.0	0.3	10.3 ± 0.2	1.5	9.9 ± 0.1	1.1
	100	97.7 ± 2.9	3.0	96.2 ± 3.4	3.6	91.7 ± 1.6	1.8	99.5 ± 2.0	2.1
Quercetin	0.8	0.7 ± 0.0	1.4	0.8 ± 0.0	4.0	0.8 ± 0.0	5.3	0.8 ± 0.1	13.2
	10	9.8 ± 0.9	9.2	9.0 ± 0.2	2.6	10.5 ± 0.1	1.0	9.6 ± 0.7	7.2
	100	101.8 ± 0.3	0.3	89.3 ± 1.9	2.1	107.8 ± 1.1	1.0	109.2 ± 1.4	1.3
Apigenin	0.8	0.8 ± 0.0	2.6	0.7 ± 0.0	1.4	0.7 ± 0.0	5.6	0.8 ± 0.1	6.0
	10	8.7 ± 0.2	2.5	9.1 ± 0.5	5.4	8.9 ± 0.6	6.3	11.0 ± 0.4	3.2
	100	87.6 ± 1.0	1.2	87.1 ± 0.9	1.0	90.2 ± 3.2	3.6	107.2 ± 2.5	2.3
Resveratrol	1.6	1.6 ± 0.1	9.0	1.5 ± 0.1	5.4	1.7 ± 0.2	9.0	1.5 ± 0.1	3.3
	20	20.7 ± 0.1	0.3	20.8 ± 1.4	6.6	20.5 ± 0.5	2.6	21.2 ± 1.0	4.8
	200	204.9 ± 5.5	2.7	197.0 ± 4.3	2.2	205.9 ± 2.5	1.2	207.5 ± 4.9	2.3

**Table 6:** The main pharmacokinetic parameters of 5 analytes in rat plasma (*n* = 6, mean ± SD).

Parameters	Veratrosine	Jervine	Veratramine	Polydatin	Resveratrol
*C*_max_ (ng/mL)	236.6 ± 155.4	515.0 ± 242.4	192.7 ± 76.5	27.1 ± 24.0	45.0 ± 39.5
*T*_max_ (h)	0.08 ± 0.00	32.00 ± 6.20	34.08 ± 23.14	0.26 ± 0.29	0.18 ± 0.17
AUC_(0 − *t*)_ (h ng/mL)	410.5 ± 182.8	22636.0 ± 9392.6	7977.5 ± 2328.1	60.4 ± 18.1	89.7 ± 49.3
AUC_(0 − ∞)_ (h ng/mL)	420.9 ± 199.4	22695.8 ± 9430.8	7996.1 ± 2329.2	71.2 ± 13.9	100.8 ± 51.5
*T*_1/2_ (h)	11.48 ± 7.04	13.56 ± 1.36	14.15 ± 6.51	14.40 ± 10.55	2.24 ± 1.11
MRT_(0‐24 h)_ (h)	10.51 ± 6.90	38.78 ± 5.58	38.78 ± 5.83	13.25 ± 2.98	13.17 ± 7.77
MRT_(0 − ∞)_ (h)	11.34 ± 7.68	39.06 ± 5.53	39.01 ± 5.91	16.48 ± 13.66	18.98 ± 15.53

## Data Availability

The data used to support the findings of this study are available from the corresponding author upon request.
